# Global perspectives on infectious diseases at risk of escalation and their drivers

**DOI:** 10.1038/s41598-025-22573-3

**Published:** 2025-11-04

**Authors:** Ryan James Walker, Paul Tunde Kingpriest, Jenny Gong, Molly Naisanga, Mir Nabila Ashraf, Javier Roberti, Trudie Lang

**Affiliations:** 1 The Global Health Network, Centre for Tropical Medicine and Global Health, Nuffield Department of Medicine, University of Oxford, Oxford, UK; 2 Gulu University, Gulu, Uganda; 3 Health Systems and Population Studies Division, Initiative for Non-Communicable Diseases, International Centre for Diarrhoeal Disease Research Bangladesh, Dhaka, Bangladesh; 4 Institute for Clinical Effectiveness and Health Policy, Buenos Aires, Argentina; 5 Epidemiology and Public Health Research Centre, National Council for Scientific and Technical Research, Buenos Aires, Argentina

**Keywords:** Public health, Epidemiology

## Abstract

Infectious disease burden is dynamic and devastating across the globe. We need to better understand and predict these threats to mitigate harm from new, re-emergent and endemic pathogens. 3,752 globally diverse participants took part in this two-step, mixed-methods adapted Delphi study. Firstly, an online survey asked health workers and researchers to identify the infectious diseases they considered to be at greatest risk of escalation in their setting, along with the factors driving this. Secondly, structured thematic workshops were hosted in Africa, Asia and Latin America, to allow in-depth exploration of the factors driving the prioritisation of these diseases. Participants considered the primary threat to be the escalation of high burden, endemic diseases, rather than emerging or re-emerging pathogen outbreaks. This was driven by the high prioritisation of vector-borne diseases (primarily malaria and dengue), tuberculosis, and HIV/AIDS. Whilst the main finding from survey responses (n = 3,700) identified growing concern over tuberculosis, participants in the subsequent workshops (n = 169) emphasised the increasing threat of vector-borne diseases. Participants considered the impact of climate change, socioeconomic factors and increasing drug resistance patterns to be driving the escalation of these diseases. This study provides striking new insight into priority infection threats due to the large scale of participation, breadth of stakeholder experience, and wide global representation. These factors allowed us to accurately determine the consensus of a substantial component of the global infectious disease research community and share unprecedented insights from the lived experience of researchers and health workers in low resource settings. We consider these perspectives particularly valuable given the absence of biological data that concurrently assesses all infectious diseases across global regions at a single point in time. Our findings represent an important new evidence-based alarm call; the next pandemic may not be a sudden event, but a slow, ‘creeping catastrophe’, impacting the most impoverished regions and communities. We need to respond now, having heard these important, consistent opinions from these previously unheard and collective voices.

**Supplementary Information:**

The online version contains supplementary material available at 10.1038/s41598-025-22573-3.

## Introduction

The world has been described as entering a ‘new era of infectious diseases’, characterised by outbreaks of emerging, re-emerging and endemic pathogens^1^. Indeed, during the last decade, the world has witnessed multiple major outbreaks and emergent new threats, from Middle East Respiratory Syndrome (MERS), to Ebola, Zika, measles and COVID-19^1,2^. At the time of writing, South America’s Southern Cone region has recently experienced an unprecedent rise in dengue cases, and a novel MPox clade has emerged on the Democratic Republic of Congo-Rwanda border, and is escalating in countries such as Seirra Leone^3,4^.

Such outbreaks continue to represent a substantial burden to healthcare systems, particularly in Low and Middle-Income Countries (LMICs)^5^. Most notably, the COVID-19 pandemic underscored the vulnerability of human society to infectious diseases, with billions in research funding subsequently directed towards pandemic preparedness to mitigate the threat of devasting future outbreaks^6,7^. In light of this ever-shifting global landscape, and with the critical need to allocate health resources effectively and equitably, we sought to understand which infectious diseases (known or novel) are currently at greatest risk of escalation, and why^8^.

Whilst biomedical data and modelling remain crucial to answering such questions, there is increasing recognition that more comprehensive, people-centred approaches to evidence generation are needed. As posited by Biehl and Petryna, sole reliance upon epidemiological studies, whilst neglecting the perspectives of the ultimate beneficiaries of interventions, is done so ‘*at the expense of better understanding and, ultimately, more meaningful and long-lasting outcomes’*^9^*.* Additionally, there is vast inequity in where infectious disease research happens, who leads, and who benefits from the evidence^10^. To tackle these concerns effectively, there is a need to increase the generation of evidence in settings from where global threats are likely to escalate, and where there is the least research capability to respond.

This study was undertaken to support the Wellcome Trust in the equitable development of their infectious disease strategy. Seeking to better understand the realities of escalating infection threats in diverse settings worldwide, Wellcome collaborated with The Global Health Network (TGHN), a global community of over 1,000,000 health researchers to learn from the experience, views and perspectives of a globally diverse body of infectious disease stakeholders^11^. This collaborative process aimed to ensure that the priority-setting was both inclusive and grounded in the real-world needs of diverse global contexts.

To achieve these aims, a robust methodological framework was applied, designed to involve diverse participants with a wide breadth of expertise. This approach was essential to capture an abundance of local views and contextual experience from contrasting perspectives. The broader goal of the study was to generate a unique body of evidence that could provide invaluable guidance for funders, governments and global health decision makers. Central to this was the need to better understand the immediate, in-situ experience of health workers and researchers across the globe, their perception and direct experience of the infection threats that they see escalating in their environments, and their views on how these threats can be most effectively addressed.

### Aims and objectives

We aimed to gain a detailed global understanding of the infection threats considered to be currently escalating (or most likely to escalate) around the globe, and the factors driving this increased risk. We aimed to achieve diverse representation in this process by recruiting participants from globally diverse roles, organisations, geographies and research interests.

## Methods

**Fig. 1:**
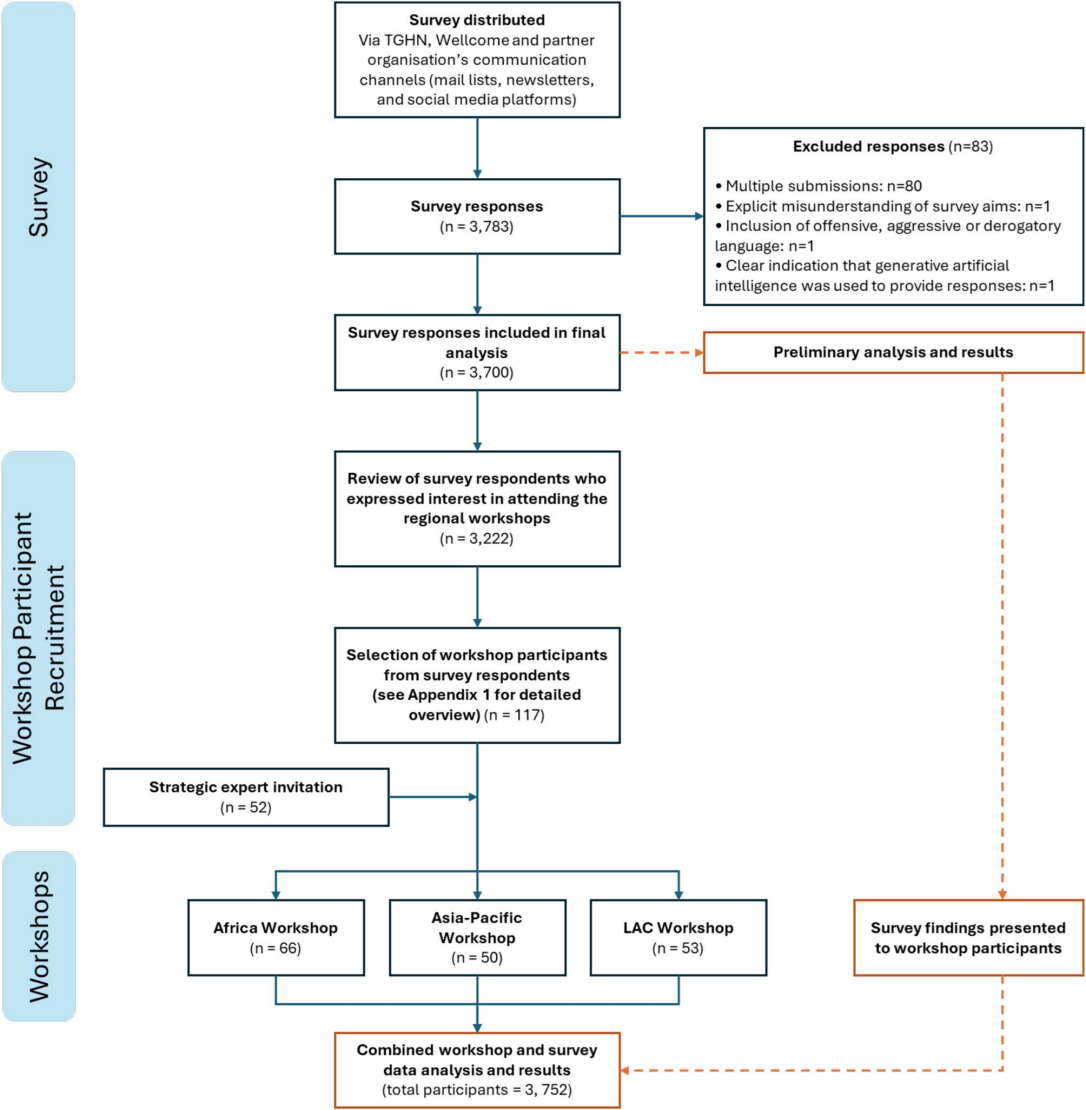
Study design and recruitment overview.

This study followed a validated, mixed-methods, crowd-based approach to consensus building, previously adapted from the Delphi technique^12–14^. An open, online, cross-sectional survey was conducted to identify the infectious diseases that participants deemed (through their experience and observations) to be the most likely, or already escalating, threat in their setting, and to provide initial insights into the contextual factors driving this escalation. Subsequently, three hybrid, Global South (Asia–Pacific, Latin America and the Caribbean [LAC], and Africa) workshops were held to elaborate upon the survey findings and build a deeper understanding of the outcomes through participant’s explanation and contextualisation of the findings. The workshops were focused on the Global South to target the regions with the greatest infectious disease burden, and the communities most vulnerable to infectious disease escalation^5^. A detailed overview of our recruitment methodology is provided in Fig. 1 and Appendix 1.

The target population was defined broadly as ‘*Anybody whose work directly impacts human health (including those working at the human-animal interface), who have research experience on understanding the sources of infectious disease and drivers of disease escalation and those involved in research uptake and public health decision-making.*’

### The survey

An online questionnaire was designed using the Jisc Online Surveys tool v3 (https://www.onlinesurveys.ac.uk/), and distributed in English, Spanish, Portuguese and French language versions. The access link was live for a three-week period in March 2023, and was disseminated via Wellcome and TGHN communications channels (mail lists, newsletters, and social media platforms). Survey participation was self-selective, and open to all individuals able to access the questionnaire hyperlink.

Participants were asked to identify the ‘*infectious disease(s), either at risk of escalation, or currently escalating’* that they considered to pose the greatest infection threat in their setting and explain why. This question was open-ended to allow participants to respond organically and reduce the impact of response bias. Participant demographic data was also collected.

### The regional workshops

Following analysis of the survey findings, the Global South workshops were undertaken to address the following aims:

1. Gain a deeper understanding of the factors driving the ranking of the highest-prioritised diseases in each region.
2. Better understand the regional infectious disease contexts and relate these to the survey findings.
3. Consider alternative interpretations of the survey findings and invite different approaches to data analysis.
4. Identify any gaps in the survey findings and explore lower-prioritised diseases.

The workshops were hosted in New Delhi (India), Rio de Janeiro (Brazil), and Addis Ababa (Ethiopia). They were closed events, with participation via invitation only, to ensure equitable and balanced participant recruitment (See Appendix 1 for a detailed overview of workshop participant recruitment). All workshop sessions were recorded (Zoom, https://zoom.us/) to enable subsequent analysis of the discussions.

Demographic data was not collected for workshop participants who had not previously completed the survey (n = 52, see Fig. 1). This was due to logistical constraints before and during the workshop.

### Exclusion criteria and data cleaning

All free-text survey responses were scanned by the research team prior to analysis. Any responses meeting the following exclusion criteria were discussed by three members of the research team:

- Explicit misunderstanding of the survey aims or terminology.
- Inclusion of offensive, aggressive or derogatory language.
- Clear indication that generative artificial intelligence was used to provide responses.

If unanimous agreement upon exclusion was reached, the entire response was excluded from the final analysis. In addition, the survey dataset was reviewed for incidences of multiple submissions by the same participant. Where identified, only a participant’s first response was included in full in the final analysis; quantitative data from repeated responses was excluded. However, qualitative data (responses to free-text questions) from subsequent submissions (that differed in content from the first response) were merged with the qualitative data from the first response and included in the final analysis.

Workshop recordings were transcribed using the voice-to-text transcription software Otter (https://otter.ai/). Each transcript was quality-checked by two researchers prior to analysis.

### Translation

Non-English free-text data (open-ended survey question responses and workshop transcripts) were translated into English prior to analysis. Spanish, Portuguese and French translation was conducted by native speakers of each language, who also possessed familiarity with the research topic. Any responses received in languages additional to those listed above were translated using Google Translate (https://translate.google.com/).

### Data analysis

Analyses were conducted for each of the following stratifications:

- Global (total of all responses).
- Economy classification (LMICs compared to High-Income Countries [HICs], as defined by the Organisation for Economic Co-operation Development Assistance Committee List of Official Development Assistance Recipients^15^. Note that the LMIC-HIC analysis was only conducted for data collected from the survey).
- Region (Asia–Pacific, Africa, LAC and the Global North).

For the regional analyses, data were divided amongst the global research team and analysed by researchers working in each respective region.

Quantitative data underwent descriptive statistical analysis using Microsoft Excel. Qualitative data underwent inductive thematic analysis using NVivo v1.5.2/13 (https://lumivero.com/products/nvivo/), guided by Braun and Clarke’s thematic analysis framework^16^. This began with an initial line-by-line review of free-text survey responses and workshop transcripts to facilitate data familiarisation. This step also allowed each researcher to independently generate a preliminary set of codes without using a predefined framework. The research team subsequently compared these preliminary codes in weekly meetings, to identify overlaps, divergences, and ambiguities, and consolidate a study-wide codebook. This was then applied to the study dataset, with regular researcher meetings to ensure its consistent application, and further refine code definitions when necessary.

To enhance inter-coder reliability, researchers independently coded the same subsets of data concurrently and then compared their results. Discrepancies were discussed, and the codebook adjusted accordingly to ensure consistency. Once all data had been coded, the research team collaboratively collated the codes into potential themes during the ongoing weekly meetings. These sessions facilitated the shared, systematic refinement of the final themes to ensure applicability to the entire dataset. All members of the research team had prior training in qualitative research methods.

When coding workshop transcripts, comments and discussion concerning the five highest-prioritised infectious diseases for each workshop region were prioritised for analysis, in line with the priority-setting nature of this study.

### Ethical considerations

The study was conducted in accordance with the Declaration of Helsinki. Ethical approval was obtained from the Oxford Tropical Research Ethics Committee (reference number: OxTREC 541–18), and all participants provided informed consent for both survey and workshop participation.

## Results

3,783 survey responses were received. Following application of the exclusion criteria and data cleaning, 3,700 responses were included in the final analysis. 169 individuals subsequently participated in the three workshops, 117 (69.2%) of which had participated in the survey. Consequently, a total of 3,752 infectious disease stakeholders participated in this study (see Fig. 1).

### Demographics

**Fig. 2:**
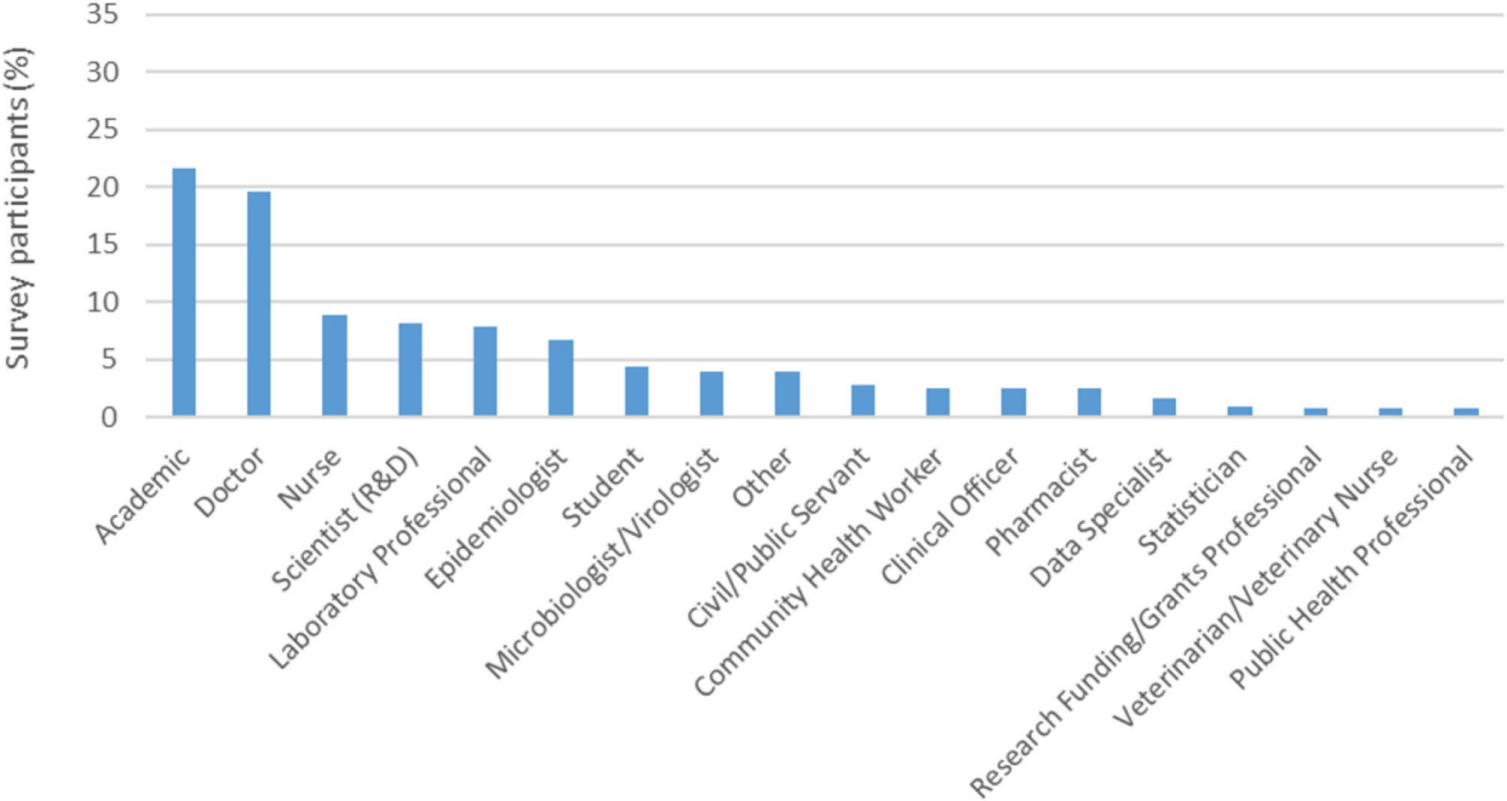
Most common professional roles of survey respondents. For readability, professional roles represented by less than 0.5% of participants are not shown. ‘Scientist (R&D)’ = Scientist (Research and Development).

Demographic data was only collected for survey participants (98.6% of total study participants, see also Methods: The regional workshops). Survey responses were received from 151 countries/territories, with 86.9% of all responses submitted by participants working in LMICs (Table 1). Respondents represented 63 different job roles, including academics, healthcare professionals, research professionals, policy/decision makers, and animal health professionals. A full overview of participant job roles is provided in Fig. 2.

**Table 1:** Overview of the national economic classification and geographic region of survey participants.

Stratification		Responses (%)
Economic Classification	LMIC	86.9%
	HIC	13.1%
	Total	100.0%
Region	Africa	48.3%
	LAC	27.2%
	Global North	12.6%
	Asia–Pacific	12.0%
	Total	100.0%*

*Cumulative percentages may not total 100.0% due to rounding.

### Greatest escalating infection threats

**Fig. 3:**
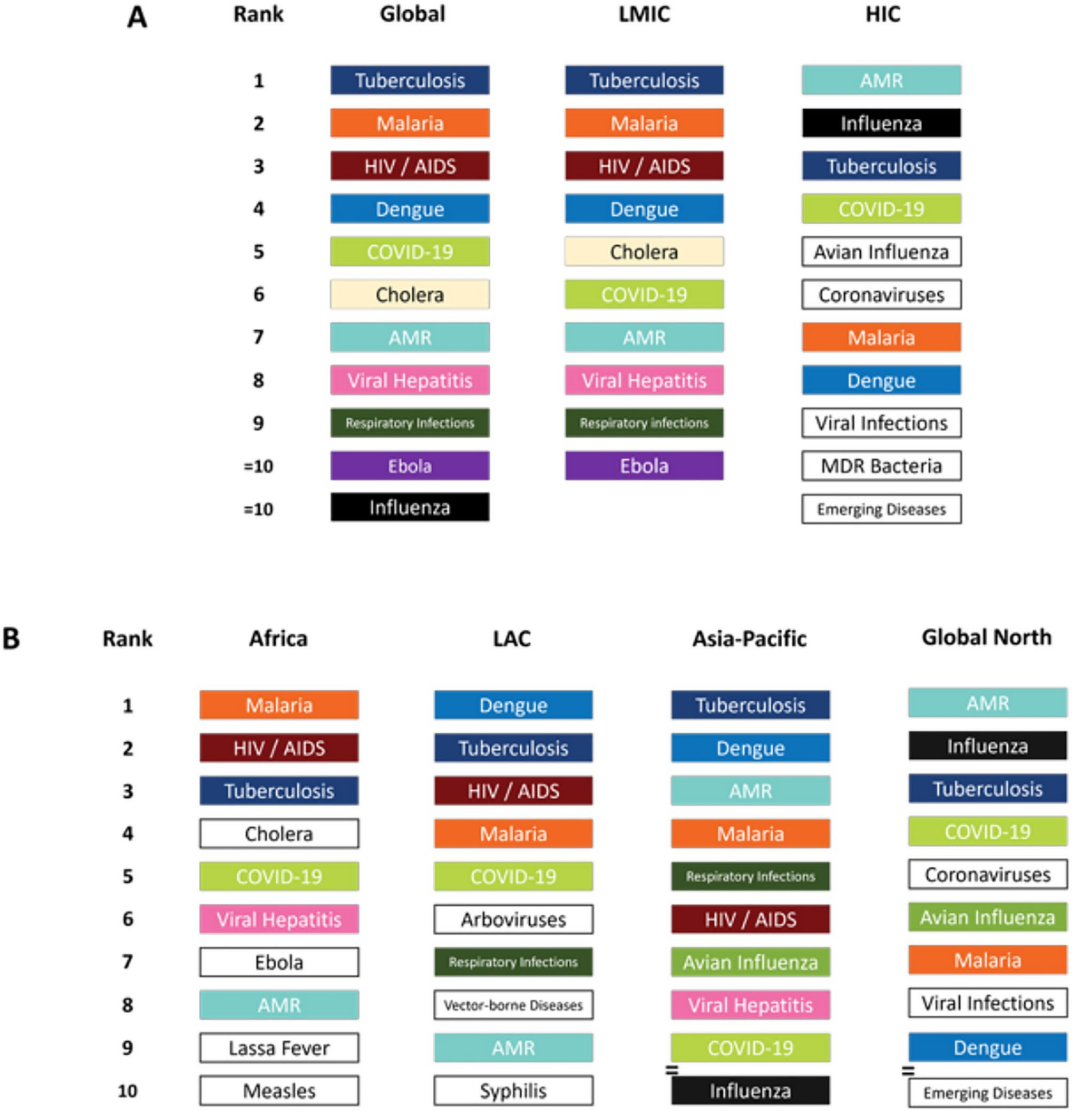
Initial survey prioritisation of diseases found to present the greatest risk of escalation. A: Global total and economic classification breakdown, B: Regional breakdown. As data was collected using a free-text box, participants were not restricted in the response options that they could provide. Hence, some participants identified specific diseases (e.g. tuberculosis, malaria, etc.), some identified disease groupings/classifications (e.g. respiratory infections, emerging diseases, etc.) and some interpreted the term ‘infection threat’ more broadly (e.g. antimicrobial resistance). In this figure we report the cumulative totals of the ‘raw’ responses provided by participants, with no researcher coding/grouping. Hence, if a participant only identified ‘Tuberculosis’ as the greatest infection threat in their setting, this was only included in the response total of tuberculosis responses and was not included in the response total for ‘Respiratory Infections’, or similar. Diseases/disease groupings that appear multiple times in each figure are colour coded for ease of comparison. ‘ = ’ indicates diseases that received the same number of responses in that stratification (e.g. in the Asia–Pacific region, COVID-19 and influenza were both identified as priorities by 13 survey participants).

An overview of the highest-prioritised infection threats from the survey is provided in Fig. 3. Initial quantitative analysis of the survey responses found that most participants perceived tuberculosis (TB) to be the disease ‘*at greatest risk of escalation, or currently escalating’* in their setting, followed by malaria and Human Immunodeficiency Virus/Acquired Immune Deficiency Syndrome (HIV/AIDS). These findings were echoed in both the LMIC and Global South regional analyses, where each of these diseases were consistently ranked within the four highest-priority infections (except for HIV/AIDS, ranked as the sixth highest-priority by participants in the Asia–Pacific region, see Fig. 3B). In contrast, whilst TB was ranked as the third greatest infection threat by participants in HICs, malaria and HIV/AIDS were deprioritised, with Antimicrobial Resistance (AMR) and influenza considered greater infection threats (Fig. 3A).

**Fig. 4:**
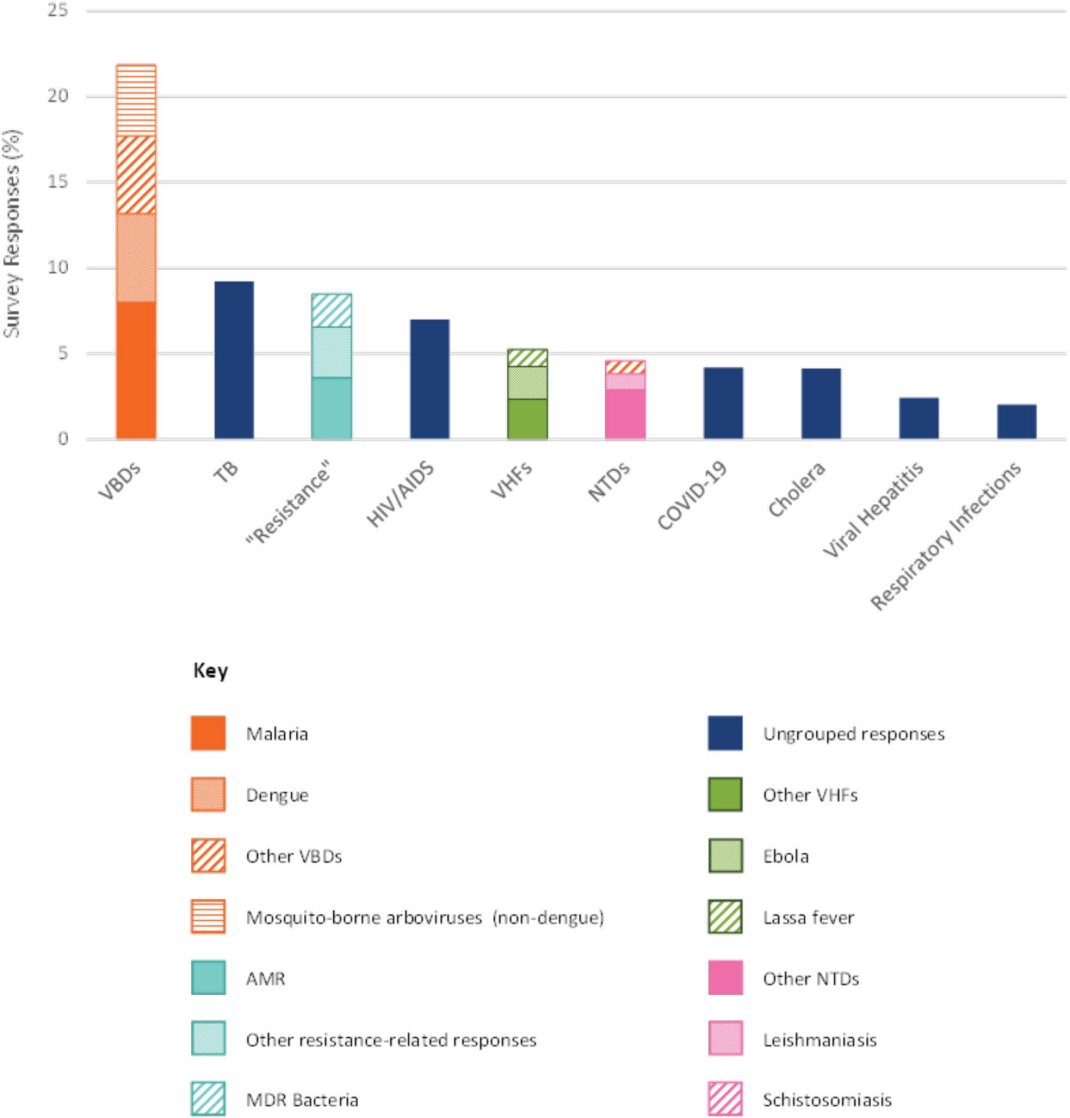
Comparison of disease groupings proposed by workshop participants, with highest-prioritised infection threats from the survey*.* For the categories ‘Resistance’, VHFs, and NTDs, the two highest-prioritised individual infection threats (e.g. Ebola and Lassa fever within VHFs) have been highlighted. ‘Ungrouped responses’ refers to infection threats featured above that have not been subjected to additional grouping (e.g. TB, HIV/AIDS, COVID-19 etc.), and represent only the total number of survey responses received for that individual infection threat. Due to the broad and diverse nature of these classifications, there is some overlap between groupings (e.g. Leishmaniasis responses, highlighted within NTDs, are also included in the ‘Other VBDs’ grouping). Dengue, highlighted in the VBD grouping, is not included in the VHF or NTD grouping. Whilst it is acknowledged that ‘severe dengue’ is considered to fall into both latter categories, as no distinction was made by any participants between dengue and severe dengue, it has not been included in the VHF/NTD categories above. MDR = Multidrug-resistant.

The second step in the methodology (the workshop) was designed to inform a secondary analysis of the survey findings. Workshop participants recommended compiling the survey responses into the following groupings for comparison; Vector-Borne Diseases (VBDs), Viral Haemorrhagic Fevers (VHFs), Neglected Tropical Diseases (NTDs) and responses relating to drug resistance. This secondary analysis found that, when considered collectively, participants considered VBDs to present the greatest threat, although TB and HIV/AIDS continued to remain highly prioritised in comparison to the other groupings/diseases (Fig. 4).

### Drivers of escalation

Thematic analysis of both free-text survey responses and the workshop discussions identified three key themes that participants considered to be driving the escalation of the prioritised diseases.

### Climate change

Participants consistently identified climate change as a major driver of the escalation of highly prioritised diseases such as malaria, dengue and cholera. Notably, climate change was considered the primary driver of VBD and arbovirus escalation across all study regions (with its impact on malaria and dengue cited in every region) due to its influence on mosquito distribution and human mobility. Other diseases where climate change was identified as a key driver of escalation across multiple regions included chikungunya (Africa, LAC and the Global North), Zika virus (LAC and the Global North) and cholera (Africa and LAC).

Beyond VBDs/arboviruses, several other disease groupings were considered climate-sensitive, with climate change identified as a key driver of escalation across multiple regions, including: zoonotic diseases (e.g. Lassa fever, leptospirosis, Japanese encephalitis, West Nile virus; all study regions), VHFs (e.g. Marburg virus, hantavirus and Crimean-Congo haemorrhagic fever; Africa, LAC and Asia–Pacific), and ‘Respiratory Diseases’ (Africa and LAC). There was consensus across every region that climate change (specifically temperature increases and changing precipitation patterns) was contributing/would contribute to the escalation of VBDs via its impact on vector distribution, including the expansion and shifting of vector ranges, increase in vector population densities, and distribution of vector breeding sites:

*“Malaria [is the greatest escalating threat as] the vectors map to temperature. Climate change is increasing the areas that vectors can survive, hence the spread of disease to areas previously not at risk”.*

*Scientist (Research and Development), Non-Governmental Organisation, Kenya*

The impact of climate change on food and waterborne infections (notably cholera and typhoid) was also discussed, primarily regarding disease escalation in Africa and the Caribbean. Driving factors centred around impact on water distribution (including water scarcity, negative impacts on water storage, and an increase in extreme weather events, specifically flooding, drought and tropical storms).

### Socioeconomic factors

Socioeconomic factors emerged as a diverse but prominent theme of disease escalation drivers. In general, concerns around socioeconomic drivers (primarily poverty, and its associated impact on habitation of informal settlements, living standards, local infrastructure capacity and unemployment) overlapped across diseases and regions. In particular, lower socioeconomic status was connected by Global South participants to lower levels of education and disease awareness in the community:

“*Tuberculosis and pneumonia [are the greatest infection threats] due to the poor economic conditions of the population and the low knowledge of the risk factors associated with transmission.”*

*Epidemiologist, Government Ministry, Honduras*

Parallels between the impact of socioeconomic drivers of disease escalation and climate change were also recognised. This included the effect on human population distribution and displacement (specifically, spurring international migration, and encroachment upon vector-endemic settings via deforestation and urbanisation):

“*Zoonotic diseases currently represent the greatest public health risk in this region, since the advance of agricultural frontiers and urbanization, together with climate change and the tropicalization of the region, increase the probability of contact with new pathogens typical or related to wildlife, generating possible spillover routes to humans.”*

*Academic, Public Health Institute, Argentina*

### AMR

Although not a disease itself, AMR was frequently identified as a priority escalating infection threat, warranting its inclusion in Fig. 3 (and the theme of drug resistance in Fig. 4). Indeed, concern regarding the impact of AMR on disease escalation was common to all study regions. AMR was cited as a driver of TB escalation across all regions, and a driver of malaria and HIV/AIDS escalation by participants in Africa and LAC. Additionally, participants raised concern about the escalating impact of AMR on gonorrhoea and nosocomial infection across all three Global South regions. Whilst multiple drivers of AMR escalation were identified, inadequate antimicrobial stewardship emerged as a recurrent theme across disease areas and study regions. Poor stewardship practices included the irrational use of antimicrobials in both human and animal populations (including overprescribing, empirical prescribing, over-the-counter dispensing, self-medication, and weak regulation).

Overlapping these concerns, discussions of nosocomial/hospital acquired infections in every workshop revealed that participants considered clinical settings to be key loci of AMR escalation. This was linked to wider, underlying health systems inadequacies including limited diagnostic capacity and a lack of AMR surveillance systems. In particular, insufficient diagnostic capability (e.g. lack of tools, facilities, and delays in accurate diagnosis) was highlighted as a driver of empirical prescribing and subsequent AMR escalation:

*“I think that with AMR, the issue is the overuse of antibiotics, and use before diagnosis or use of culture... so the issue is the under use of diagnostics, overuse of the antibiotics. If you have under use of diagnostics, what healthcare professionals see is swept under the carpet, and no one can pick it up*.”

*Academic, Health Research Institution, Thailand.*

## Discussion

The standout finding of this study is that a substantial component of the global infectious disease research community considers that the infectious diseases at greatest risk of escalation are not emerging or novel diseases at risk of sudden acceleration, but well-established, high burden diseases.

The diseases most consistently prioritised in this study (TB, malaria and HIV/AIDS) are amongst the leading contributors to global communicable disease mortality and burden^17^. However, a decline in the global prevalence, incidence, mortality rate and disease burden was observed for each of these infections between 2010–21 (with the exception of HIV/AIDS, which saw a prevalence increase of 17.5% per 100,000)^18–20^. Given that these recent epidemiological trends indicate a global ‘de-escalation’ of these infections, it is striking that participants considered these diseases to be most ‘*at risk of escalation, or currently escalating’*.

Similarly, whilst several diseases classifiable as being at high-risk of causing a Public Health Emergency of International Concern (according to the World Health Organization R&D Blueprint for Epidemics) were highly prioritised in every region (i.e. Africa: cholera, COVID-19, Ebola, Lassa fever; LAC: COVID-19; Asia–Pacific: COVID-19, influenza; Global North: influenza, coronaviruses, avian influenza), in the Global South, they consistently remained secondary to TB, malaria and HIV/AIDS^21^. Hence, whilst consensus amongst Global North participants was less defined, participants from the Global South considered the primary threat to be the escalation of high burden, endemic diseases, rather than emerging/re-emerging pathogen outbreaks. We consider this particularly notable given the timing of this study, occurring only two years after the outbreak of one of the deadliest pandemics in human history (COVID-19), itself the consequence of an emergent pathogen^22^.

The contrast of our findings with global epidemiological trends appears to be driven by concerns that three key factors, namely climate change, ongoing shifts in socioeconomic patterns, and increasing levels of AMR, are driving, or have the potential to drive, escalation of these endemic diseases.

Climate change emerged as a key driver of concern for VBDs. Participants reported that they had witnessed climate change altering mosquito distribution and human mobility patterns, contributing to the emergence, re-emergence, or increased incidence of diseases such as malaria, dengue, chikungunya, and Zika in new areas. Indeed, when considered collectively, VBDs, emerged as a disease group of extreme concern (Fig. 4). This was driven by the high prioritisation of malaria in Africa, LAC and Asia–Pacific, and dengue in LAC and Asia–Pacific (Fig. 3). Participants’ call to consider VBDs as a distinct, priority disease group reflected a shared view that disease escalation is best addressed through vector-focused, rather than disease-specific, strategies.

Participants highlighted how the interplay between climate-related and socioeconomic drivers (for example, shifting agricultural frontiers and urbanization) will dictate where the burden will fall from these climate driven risks. This was the same for both TB and AMR, where consistent comments expressed how the lowest income communities experience the greatest burden. Many participants also commented that furthering our collecting understanding through similar priority-setting studies is critical to identifying and assessing infection threat mitigation strategies. Participants also called for action, stressing the need to act immediately against these social drivers to be better prepared and protect against future pandemics. This is evidenced by the following quote from this discussion theme; ‘*’Better understanding of the social drivers of transmission of respiratory diseases could have been crucial during COVID*’’.

We consider the finding that high burden, endemic diseases of poverty are considered to pose the greatest infection threats, rather than novel or emerging pathogens, meaningful new evidence, and the outcome was surprising to the investigators and wider collaborators in this study. Does this highlight the failure of existing mitigation efforts targeting these diseases, and waning focus and interest from the global health community? This finding also highlights how if we solely rely on epidemiological data (e.g. the declining global prevalence, incidence, mortality rate and disease burden of these high burden diseases) then larger, globally diverse perspectives, and a very different lens of our knowledge and understanding of current disease threats, would be missed. To keep this engagement, enable such dialogue, and expand our ability to generate this lived-experience evidence, we need to support and value the networks and research communities that drive this important form of research.

The participants in this study represented a broad spectrum of roles and experience, from academics to healthcare workers, laboratory professionals, government representatives, research funders and animal health experts. The large scale of participation (3,752 stakeholders) represents an unprecedented level of inclusion in a health research priority setting activity, in comparison to previous initiatives^21,23–25^. Moreover, strong engagement from LMICs (86.8% of participants) ensured meaningful representation from regions that are typically underrepresented in health research priority setting activities^26^. We therefore believe that these findings present a representative body of perspective from a diverse cross-section of the global infectious disease stakeholder community.

### Limitations

A significant constraint of this study stems from the uneven distribution of data, with the African region contributing to approximately half of the survey responses, and the greatest proportion of workshop participants (Fig. 1**, **Table 1). Despite the broad geographical distribution of participants, this disproportionate representation is likely to have influenced the study outcomes, and should be considered when interpreting the collective, or ‘global’ findings. To mitigate this, we encourage readers to place particular focus on the outcomes of the stratified analyses (with emphasis on the regional breakdowns) to support more accurate understanding of regional trends in disease prioritisation.

In addition, we wish to emphasise that our findings represent the opinions of a diverse global community of infectious disease stakeholders and should not be interpreted as empirical evidence on disease burdens or transmission patterns. We acknowledge that a diverse range of factors (training and education, personal research interests, successes/failures of previous health research initiatives, mass media, funding, etc.) may have influenced participants’ responses, and that some findings may reflect the influence of these factors, rather than accurate, ‘on-the-ground’ realities. Nevertheless, we urge that these insights be valued for what they offer; an unparalleled view into the lived experiences and perceptions of those engaged in infectious disease control worldwide. Considered alongside empirical data, they provide a more holistic, people-centred understanding of escalating infectious disease threats.

Finally, due to the large scale of participation, this study generated a substantial dataset, to the degree that it has not been possible to report all outcomes here. This rich data source presents many opportunities for further exploration, including alternative disease groupings/data stratifications, detailed analysis of the barriers and enablers of infectious disease research, and a deeper exploration of the unexpected or outlier findings. We encourage others to engage with the dataset and generate new insights from alternative perspectives.

## Conclusion

Whilst there remains the possibility of a new pathogen emerging anywhere in the world, our results reveal a consensus that the next pandemic might not be a sudden event but could creep up as a slowly-building humanitarian disaster, as the catastrophic burden of endemic diseases escalates and hits new, vulnerable communities across different geographies. Therefore, we need to be aware and ready, everywhere; asking, listening, measuring and observing, to identify any escalating threats and produce new evidence to understand them. This work provides important corroborated warnings from the lived experience of researchers and health workers across the world, that should alert us as to how climatic events, socioeconomic factors and patterns of resistance could cause a devastating rise in high burden diseases.

Participants identified similar drivers of disease escalation (climate change, socioeconomic drivers, and drug resistance) regardless of setting, disease area or pathogen type. We consider this the positive news from this work, as this suggests that a systematic, cross-cutting approach to addressing these drivers could collectively tackle these long-standing infection threats. Furthermore, addressing these disease-agnostic drivers would mitigate the impact of both endemic diseases of poverty, and emergent new threats.

This study reports the perspectives of 3,752 stakeholders from diverse backgrounds, locations and areas of expertise. Analysis of their perspectives has provided deeper context and clarity on where the greatest threats are deemed to lie. Our inclusive approach, and the volume of participants, made it possible to robustly examine this unprecedented number of observations in detail, and conclude that VBDs (primarily malaria and dengue), alongside TB and HIV/AIDS, are the most pressing and escalating infection threats observed by researchers and healthcare workers in low resource settings, with climate change, socioeconomic factors and increasing drug resistance as the major drivers. These perspectives provide a new understanding of the factors driving global vulnerability to infectious diseases, and insight into how this risk is being compounded by issues such as poverty, population distribution and climate change. The diseases of greatest concern are well-established, high burden diseases. Not only do they remain pervasive, but they represent an increasing threat – a slow, ‘creeping catastrophe’ – for which we need mechanisms in place, everywhere, to identify where they are appearing or reappearing, and maintain a strong pipeline of new interventions for both prevention and treatment.

We believe we should take heed in the experience, observations and opinions shared by participants in this study. This vast, collective voice called out what we were not expecting; they consider that endemic diseases of poverty represent a greater threat than an emergent unknown. Importantly, their identification of common factors driving the escalation of these diseases offers the global health community a tangible opportunity to develop new, cross-cutting strategies to tackle not only entrenched diseases of poverty, but emergent new threats.

## Supplementary Information

Supplementary Information.

## Data Availability

The dataset for this work is publicly available at- https://ora.ox.ac.uk/objects/uuid:4ad23d94-d4da-4e31-8d2d-7b4960bb1d3c with the 10.5287/ora-xoybqx4yj.
