# Supplementary material for: Global perspectives on infectious diseases at risk of escalation and their drivers
Source: Sci Rep. 2025 Nov 4;15:38630. doi: 10.1038/s41598-025-22573-3 (PMC12586545; doi:10.1038/s41598-025-22573-3)
Supplement: Supplementary file 1 — Supplementary Information. [file 41598_2025_22573_MOESM1_ESM.pdf]

Appendix 1: Workshop recruitment methodology

Overview
A dual approach was used to recruit workshop participants. Primarily, participants were invited from the pool of survey respondents that had expressed a desire to be considered for workshop participation (n=3,222). This was to capitalise on the diversity of survey participants and ensure wider, inclusive, and globally representative involvement in the priority-setting discussions. Additionally, strategic invitations were made to field experts (in both human and animal health) through Wellcome and The Global Health Network's extended networks of collaborators and partner institutions, to ensure expert contribution to the consensus building discussions.

Following consideration of logistical factors (venue size, travel logistics, time constraints etc) a maximum target of 75 individuals (including researchers and support staff) per workshop was agreed.
Recipients of strategic invitations, and research and support staff for each workshop, were determined first to establish the total number of available spaces remaining for survey participants at each workshop. Next, using the selection method outlined below, provisional lists of potential workshop participants from the pool of interested survey respondents were produced. The number of potential participants proposed on each list was double that of the available spaces for survey participants at each workshop (i.e. [75 – total number of strategic invitees, research and support staff]x2), to allow for negative or non-responses to the workshop invitations.

Invitation emails were then distributed to all potential participants (both strategic invitations, and survey respondents, starting May 2023), and responses collected over a week-long period. Each invitee also received a reminder email during this period. Following this period, if not all the available 75 places at each workshop had been filled, the survey-participant selection process was repeated, to produce additional potential participant lists to fill the remaining gaps. This process was repeated further if necessary.

Multistage Randomisation with Diversity Testing and Free Text Review
A rigorous selection strategy was developed to ensure that the following three criteria were satisfied when selecting workshop participants from the survey respondents:

- **Engagement**: Participants would be willing and able to contribute actively to in-person workshop discussions.
- **Fairness:** Participants were selected in a fair, unbiased manner.
- **Diversity**: Participants represented a wide and representative pool of stakeholders working within infectious disease research.

This strategy, dubbed Multi-Stage Randomisation with Diversity Testing and Free-Text Review (MSR-DT-FT), is outlined in detail in **Appendix 1 Figure 1.**

Firstly, only participants that expressed an interest in workshop participation during the survey were considered for workshop participation. From the subsequent pool of responses, all participants that provided no written response to at least one of the three, primary, open-ended survey questions (regarding diseases posing the greatest infection threats, the types of research most urgently needed, and the barriers and enablers to research in this field) were excluded, under the rationale that those who had provided written responses:

- Were more likely to be communicative in a workshop environment.
- Had demonstrated greater interest and investment in the study by committing time to written responses.

- Could be proved to demonstrate a clear understanding of the research subject, as opposed to individuals who only selected pre-existing response options to close-ended questions.

The above was deemed to satisfy the 'engagement' criteria of the selection process.

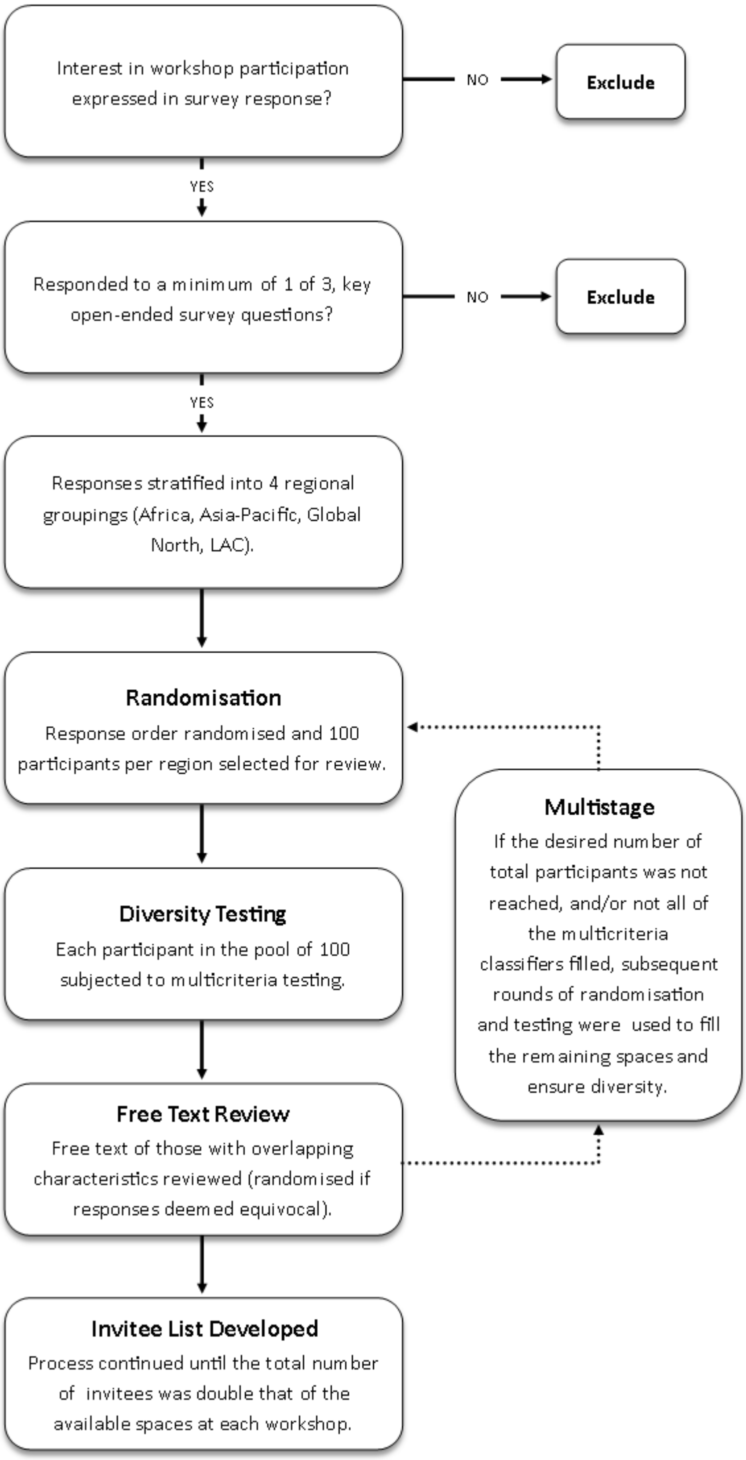

Appendix 1 Figure 1: Overview of the workshop participant recruitment process

From this subsequent pool of responses, the data was stratified to provide response sets for each of the four study regions (Africa, Asia-Pacific, Global North and LAC, see below). Within each of these regional response sets, the response order was then randomised (satisfying the 'fairness' criteria), and the first 100 randomised responses subjected to multicriteria testing and free text review, to satisfy the final criteria of the selection process, 'diversity'.

A multicriteria test was developed based on participant demographic data (see **Appendix 1 Figure 2**), which was applied sequentially to each participant included in the randomised pool. Following the selection of the first participant, the demographic details of each subsequent potential participant were compared to those previously selected. If the 'first criteria' details (Type of Research Experience) were found to overlap, then the 'second criteria' details (Disease Area of interest) would then be compared, and so on, until discrepancies were identified to justify the potential participant's selection. If all demographic details of a potential participant were found to feature across the pool of previously selected participants, that individual was excluded from the current round of selection.

In incidences where potential participants shared the same demographic details, or when a decision could not be reached between two or more potential participants using the multicriteria test, the free-text responses of the participants in question were then reviewed (free text review). Participants deemed to have given considered, well-structured and insightful responses were favoured for selection (in alignment with the 'engagement' criteria). In such incidences, the free-text responses in question were discussed amongst three members of the research team before a final decision on participant selection was made.

If a decision on participant selection could still not be made (for example, due to a similar quality of free-text responses), randomisation was used to make a final selection from the participants in question.
This above selection process was used to produce invitation lists for each workshop. The process continued until the total number of invitees on each list was double that of the available spaces for survey participants at each workshop (see **Appendix 1 Overview**).

Global North Participation
As the workshops were only held in the three Global South study regions (Africa, Asia-Pacific, LAC), Global North workshop invitees were allocated to one of the three Global South invitation lists. A target of 33.3% of Global North survey participants was aimed for across each of the lists, to ensure Global North representation at each workshop.

Decisions on which workshop to invite individual Global North participants to were made pragmatically, following consideration of language, geography, shared regional healthcare agencies / organisations (e.g. PAHO; LAC and Northern America), participant research experience/background, and available workshop spaces.

Multicriteria Test
A multicriteria test was applied to ensure diversity of representation amongst the workshop participants, to gather experience and perspectives from across the entirety of the infectious disease research ecosystem.

As a result of the study's ultimate focus on research funding priorities, the test sequence was structured to prioritise diversity of participant research experience and disease areas of interest over other demographic criteria. This was achieved by making 'Type of Research Experience' and 'Disease Area of Interest' the first two criteria in the test. This approach was deemed necessary to ensure holistic representation of individuals engaged across all types of research infectious disease research.

Participant gender information was not gathered during the survey, however the importance of ensuring gender diversity amongst workshop participants (to ensure gender-equitable contribution to the consensus-building discussions and study outcomes) was noted during the participant selection process. Gender was therefore included as an additional component of the multicriteria test, in an effort to ensure gender balance at the workshops.

The approach to determining participant gender was guided by previous peer-reviewed approaches to inferring gender identity.[1,2] A Google search for each potential participant was conducted (using participant name as a search term), and publicly-available information (pronouns, photographs, titles etc) screened by 3 researchers to infer gender identity. As this process was designed to serve as a general guide to ensuring gender diversity amongst the workshop participants, and given the fact that participants would have the opportunity to self-identify their gender during the workshop further approaches to participant gender determination (for example, the use of specialised web applications) were deemed unnecessary at this point.

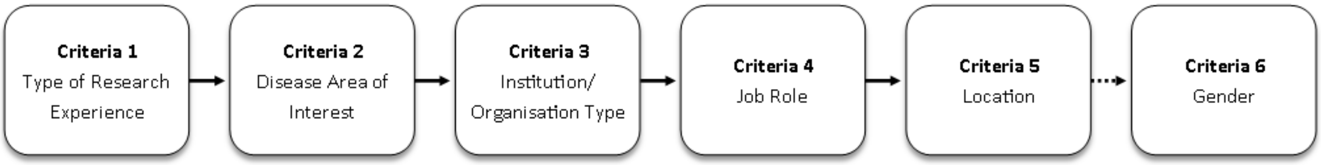

Appendix 1 Figure 2: Structure of the 'Multicriteria Test' component of the recruitment process. Criteria 1-5 were assessed using participant survey response data. If consideration of Criteria 6 (Gender) was necessary, it was assessed using the method described above.

1. van Daalen KR, Chowdhury M, Dada S, Khorsand P, El-Gamal S, Kaidarova G, Jung L, Othman R, O'Leary CA, Ashworth HC, Socha A, Olaniyan D, Azeezat FT, Abouhala S, Abdulkareem T, Dhatt R, Rajan D. Does global health governance walk the talk? Gender representation in World Health Assemblies, 1948-2021. BMJ Glob Health. 2022 Aug;7(8):e009312. doi: 10.1136/bmjgh-2022-009312. PMID: 35998979; PMCID: PMC9403126.
2. Global Health 50/50. Boards for all? A review of power, policy, and people on the boards of organisations active in global health. 2022. www.globalhealth5050.org/2022-report.
